# Nivolumab Induced Adrenal Insufficiency: Rare Side-effect of a New Anti-cancer Therapy - Immune-checkpoint Inhibitors

**DOI:** 10.7759/cureus.7625

**Published:** 2020-04-10

**Authors:** Maitreyee Rai, Mylene Go

**Affiliations:** 1 Internal Medicine, Crozer Chester Medical Center, Upland, USA; 2 Hematology Oncology, Crozer Chester Medical Center, Upland, USA

**Keywords:** immune-checkpoint inhibitors, immune-related adverse events, nivolumab-induced adrenal insufficiency, adrenal insufficiency, hypophysitis, nivolumab

## Abstract

Immune-checkpoint inhibitors are immuno-modulatory antibodies used in patients with advanced cancers like melanoma, renal cell carcinoma, non-small cell lung cancer, etc. They are associated with a wide array of side effects, commonly known as immune-related adverse events (irAEs), affecting dermatological, gastrointestinal, hepatic, endocrine, and other systems. We present a case of nivolumab-induced adrenal insufficiency in a patient presenting with refractory hypotension. The patient is a 77-year-old caucasian male with metastatic renal cell carcinoma (RCC) on nivolumab therapy, presented to his primary doctor for symptoms of fatigue, weakness, loss of appetite, and dizziness. His initial blood pressure (BP) was noted to be 78/44 mmHg, so he was referred to the emergency department. He received several liters of intravenous (IV) fluid boluses; however, BP consistently stayed in 90s systolic and 40-50 diastolic. The lab investigations showed a low sodium level at 128 mmol/L, blood urea nitrogen (BUN) elevated at 37 mg/dL, creatinine elevated at 2.7 mg/dL. A morning cortisol level was checked; it came back low at 1.3 mcg/dL. Further testing with the cosyntropin stimulation test revealed low basal cortisol of 1 mcg/dL and only a mild increase to 10.20 mcg/dL after the cosyntropin administration. Adrenocorticotrophic hormone (ACTH) was checked that came out to be low <5pg/mL, favoring a diagnosis of secondary adrenal insufficiency likely due to hypophysitis. In the meantime, the patient was started on hydrocortisone, which improved his blood pressure significantly. He was eventually weaned from IV hydrocortisone to p.o. hydrocortisone. The nivolumab was discontinued, and oncology decided on giving a nivolumab re-challenge once the patient was stabilized. Our patient presented with common manifestations of adrenal insufficiency like fatigue, hypotension, and hyponatremia, which is one of the rare irAEs occurring in <1% of the patients. These are non-specific manifestations and can be easily overlooked if adverse events of immunotherapy are not suspected. Even though rare, adrenal insufficiency is a life-threatening side-effect of immune checkpoint inhibitor drugs that need to be recognized immediately and managed with intravenous glucocorticoids.

## Introduction

Recent advances in cancer research have lead to the development of immune-checkpoint inhibitors that are immuno-modulatory antibodies targeting: programmed cell death receptor-1 (PD-1) [e.g., nivolumab, pembrolizumab], or programmed cell death ligand-1 (PDL-1) [e.g., atezolizumab, avelumab], or cytotoxic T-lymphocyte-associated antigen 4 (CTLA-4) [e.g., ipilimumab] [1, 2]. These have been recently approved to be used in patients with advanced cancers like melanoma, renal cell carcinoma, non-small cell lung cancer, etc. However, checkpoint inhibition does come with a wide array of side effects, commonly known as immune-related adverse events (irAEs), affecting dermatological, gastrointestinal, hepatic, endocrine and other systems [1, 3]. Pertinent to our case, nivolumab is known to be more commonly associated with thyroid dysfunction, and rarely causes hypophysitis (<1%) or adrenal insufficiency (0.7% of patients in randomized clinical trials) [1, 3-5]. We present a case of nivolumab-induced adrenal insufficiency in a patient presenting with refractory hypotension. 

## Case presentation

The patient is a 77-year-old male with a past medical history of renal cell carcinoma (RCC) status post right nephrectomy, now metastatic to the lungs, syndrome of inappropriate anti-diuretic hormone (SIADH), hypertension and congestive heart failure (CHF). He presented to his primary doctor for symptoms of fatigue, weakness, decreased appetite and dizziness. The review of systems was negative for any fever, chills, chest pain, palpitations, cough, shortness of breath, diarrhea, hematochezia or melena, dysuria, polyuria, polydipsia, tremors, heat or cold intolerance. No history of trauma or apparent blood loss was evident. He reported being on immunotherapy with nivolumab for his metastatic renal cell carcinoma. He had been on nivolumab for the last six months, and the current symptoms started after his last dose, which was two weeks ago.

The patient's initial blood pressure (BP) in the doctor's office was noted to be 78/44 mmHg, so he was referred to the emergency department (ED). On arrival in the ED, his BP was 96/50 mmHg, heart rate (HR) 72 beats per minute, and body temperature 97.3° F. He received several liters of intravenous (IV) fluid boluses; however, BP consistently stayed in 90s systolic and 40-50 diastolic. His physical exam was significant for known chronic bilateral lower extremity pitting edema; heart sounds were heard normal S1, S2 with regular rate and rhythm, no murmurs or rubs or gallops, no jugular venous distension. Lung sounds were heard clear, normal vesicular** **breath sounds were bilateral, no wheezes, crackles, or rhonchi. The skin was warm to touch, with no rashes or open wounds. The abdomen was soft, non-tender, no visible or palpable organomegaly, bowel sounds were heard normal. The lab investigations (Table 1) was significant for a white blood cell count (WBC) of 4.0 u/L (normal 4.8-10.8 x 10*3/uL), low sodium (Na) level at 128 mmol/L (decreased from his baseline of 133-139 mmol/L, normal 135-146 mmol/L), blood urea nitrogen (BUN) elevated at 37 mg/dL (normal 10-20 mg/dL), creatinine of 2.7 mg/dL (elevated from his baseline of 1.1-1.4 mg/dL, normal 0.6-1.1 mg/dL). His troponins were not detectable. EKG did not reveal any ST - T segment changes suggestive of new ischemic changes. He was initially started on empiric broad-spectrum antibiotics in view of possible sepsis. Antibiotics were eventually discontinued since there were no evident sources of infection and a lack of fever or leucocytosis, making sepsis as the cause for hypotension less likely. Chest X-ray was negative for any pulmonary consolidation, infiltrate, effusion, pneumothorax, or mediastinal shift. Urine and blood cultures were negative. His probability of pulmonary embolism was low with a low Wells score of 1, secondary to his history of malignancy. He did have a history of congestive heart failure; however, the patient denied any chest pain or shortness of breath or orthopnea and did not examine as being volume overloaded, cool clammy skin, any worsening heart murmur, findings suggestive of worsening CHF or new cardiac ischemia or valvular dysfunction as a cause for his hypotension. A transthoracic echocardiogram showed an ejection fraction of 25%, same as his baseline, no acute valvular dysfunction, no right heart strain or pericardial tamponade. In view of the complaints of hypotension not responsive to fluid boluses, a morning cortisol level was checked to look for possible adrenal insufficiency. It came back low at 1.3 mcg/dL (normal 10-20 mcg/dL). Further testing with the cosyntropin stimulation test (Table 1) revealed low basal cortisol of 1 mcg/dL and only a mild increase to 10.20 mcg/dL after the cosyntropin administration. These findings were suggestive of adrenal insufficiency. In order to differentiate whether it’s primary or secondary adrenal insufficiency, adrenocorticotrophic hormone (ACTH) level was checked that came out to be low < 5 pg/mL, favoring a diagnosis of secondary adrenal insufficiency. 

**Table 1:** Laboratory results

Laboratory studies	Results
Sodium (Na) [normal 135 - 145 mEq/L]	128 mEq/L
Blood urea nitrogen (BUN) [normal 7 - 20 mg/dL]	37 mg/dL
Creatinine (Cr) [normal 0.6 - 1.2 mg/dL]	2.7 mg/dL
Morning (AM) cortisol levels [normal 10 - 20 mcg/dL]	1.3 mcg/dL
COSYNTROPIN STIMULATION TEST	
Basal cortisol	1 mcg/dL
Cortisol level after cosyntropin administration [normal >/= 18 - 20 mcg/dL]	10.2 mcg/dL
Adrenocorticotrophic hormoone (ACTH) level [normal 10 - 50 pg/mL]	<5 pg/mL

**Figure 1:**
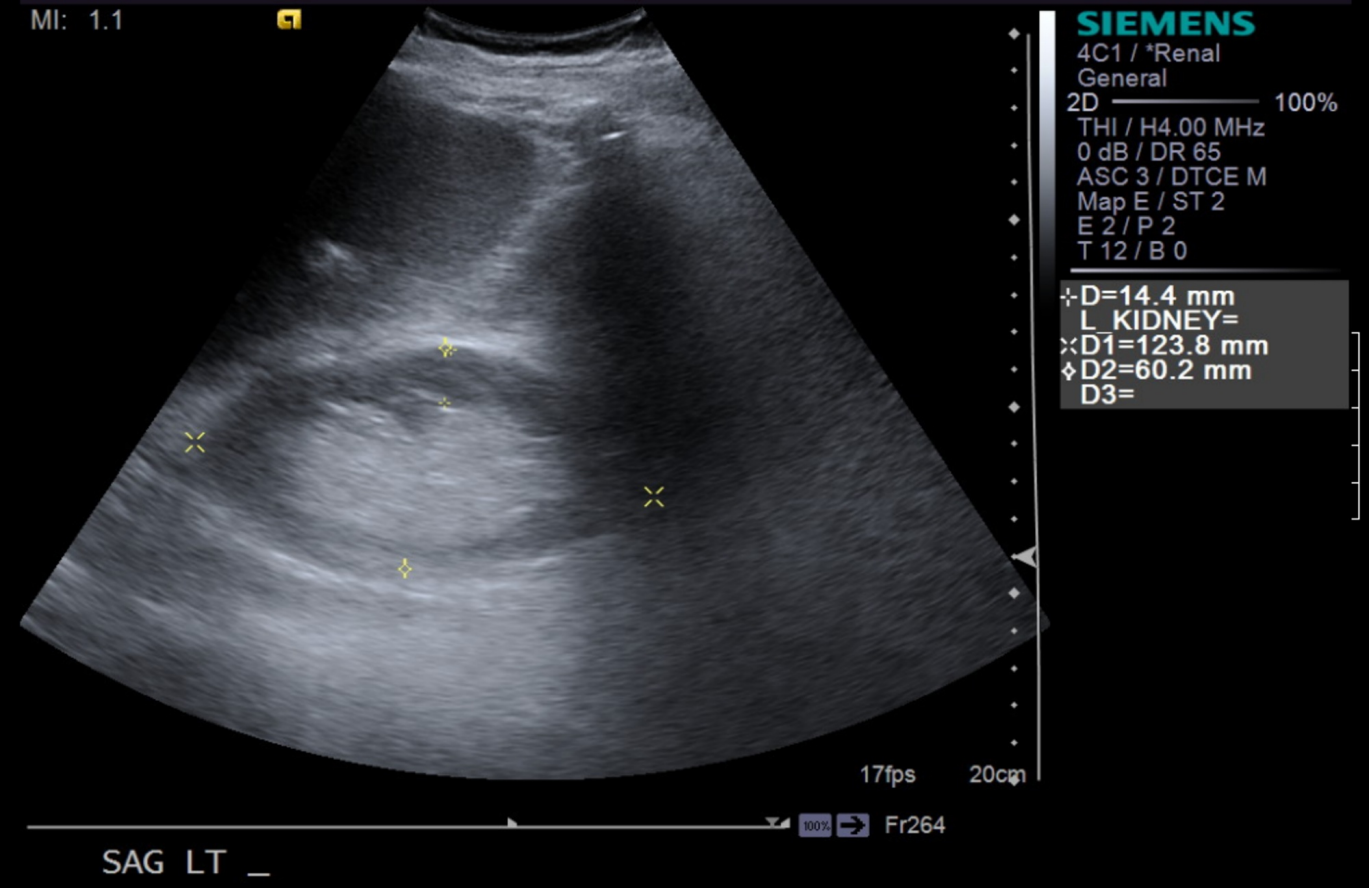
Ultrasound image of the left kidney (yellow asterisk)

Endocrinology was consulted, who determined that it is most likely hypophysitis secondary to nivolumab use, which is known to cause multiple endocrinopathies. The thyroid hormone panel was checked that revealed thyroid-stimulating hormone (TSH) and thyroxine (T4) levels to be within normal limits. In the meantime, the patient was started on intravenous hydrocortisone, which improved his blood pressure significantly. He was eventually weaned from IV hydrocortisone to p.o. hydrocortisone and discharged on the same. Additionally, the patient had an acute kidney injury on top of his chronic kidney disease due to hypotension and volume depletion in the setting of adrenal insufficiency. A left renal ultrasound done revealed findings of chronic medical renal disease (Figure 1). It resolved after fluid resuscitation and IV hydrocortisone. Also, the significant hyponatremia on admission, was likely secondary to his history of longstanding SIADH, worsened by his new-onset adrenal insufficiency. It resolved when the patient was started on hydrocortisone and salt tablets. The nivolumab was discontinued, and oncology decided on giving a nivolumab re-challenge once the patient was stabilized. 

## Discussion

**Figure 2:**
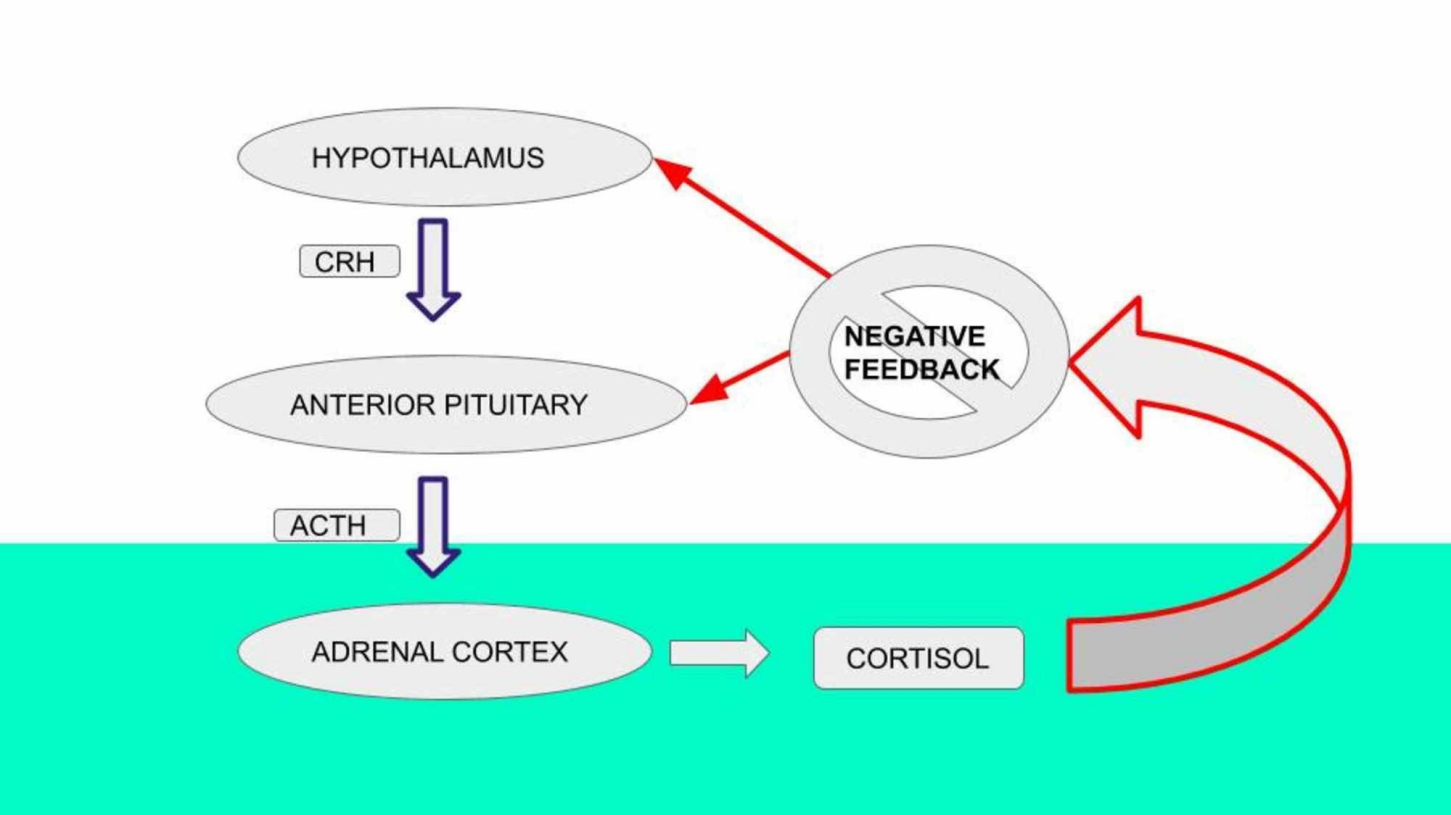
Hypothalamic-pituitary-adrenal axis CRH - corticotropin-releasing hormone; ACTH - adrenocorticotrophic hormone

Our patient presented with common manifestations of adrenal insufficiency like fatigue, hypotension, and hyponatremia, which is one of the rare immunotherapy related adverse events (irAEs) occurring in <1% of the patients, usually presents two months after the initiation of therapy [1, 6, 7]. These are non-specific manifestations and can be easily overlooked if adverse events of immunotherapy are not suspected. The following image illustrates the hypothalamic-pituitary-adrenal axis function (Figure 2) [8].

Disruption in hormone production at any level (pituitary or adrenal) leads to decreased cortisol production and hence, to adrenal insufficiency. As in our patient, the low ACTH and low cortisol levels are consistent with secondary adrenal insufficiency. 

The use of immune checkpoint inhibitors (ICPIs) blocks the immune checkpoint molecules like PD-1, PDL-1, and CTLA-4, these molecules are primarily involved in damping down the immune reaction to self-antigens and hence results in immunologic tolerance. By inhibiting these immune checkpoint molecules, the ICPIs lead to an increase in the baseline T-cell-specific immune response that turns the immune system against the tumor [9]. However, this process can also result in an unchecked immune response that manifests clinically in the form of numerous autoimmune-like/inflammatory adverse effects. Examples of such adverse effects include dermatologic, hepatic, gastrointestinal, endocrine (pituitary, adrenal), renal, and pulmonary toxicities. Of these, the most serious ones can include adrenal crisis, hypophysitis, Stevens-Jonson syndrome, toxic epidermal necrosis, colitis, etc. [2].

## Conclusions

Even though rare, adrenal insufficiency is a life-threatening side-effect of immune checkpoint inhibitor drugs that need to be recognized immediately and managed with intravenous glucocorticoids. With the increased use of these drugs in the management of advanced cancers, there is an increased number of patients presenting to the offices or emergency departments with complications secondary to irAEs. Therefore, as an internist, it’s imperative to be able to diagnose and intervene at an early stage to prevent serious outcomes that may result in the discontinuation of effective anti-cancer therapy.
